# Editorial: Insights into structural and functional organization of the brain: evidence from neuroimaging and non-invasive brain stimulation techniques

**DOI:** 10.3389/fpsyt.2023.1225755

**Published:** 2023-06-12

**Authors:** Masaru Tanaka, Matteo Diano, Simone Battaglia

**Affiliations:** ^1^ELKH-SZTE Neuroscience Research Group, Danube Neuroscience Research Laboratory, Eötvös Loránd Research Network, University of Szeged (ELKH-SZTE), Szeged, Hungary; ^2^Department of Psychology, University of Turin, Turin, Italy; ^3^Center for Studies and Research in Cognitive Neuroscience, Department of Psychology “Renzo Canestrari”, Cesena Campus, Alma Mater Studiorum Università di Bologna, Cesena, Italy

**Keywords:** substance-related disorders, bipolar disorder, dysmenorrhea, dyspepsia, schizophrenia, biomarkers, machine learning

The brain is a complex and dynamic system that underlies our behavior, emotions, and cognition (1–3). To better understand the structural and functional organization of the brain, neuroimaging and brain stimulation techniques have emerged as powerful tools (Nyatega et al.) (4–9). The development of non-invasive brain stimulation (NIBS) techniques has substantially enriched our understanding of human brain function across the last decades (10, 11). An increasing number of studies have used different NIBS protocols in various research disciplines, spanning electrophysiological applications (12), studies of human cognition (13, 14), physiological markers (15, 16) and the treatment of neurological and psychiatric disorders (17). These techniques allow researchers to investigate the brain's underlying mechanisms and neural networks in real-time, enabling new insights into the diagnosis and treatment of neuropsychiatric disorders: while neuroimaging provides correlational evidence for structure–function relationships, NIBS provide causal relevance of a given brain region for a function of interest, but also the interaction between several nodes in larger brain networks (18). Recent advances in dynamic functional connectivity have expanded our ability to probe and understand the interplay among brain regions and their responses to TMS. By detecting and analyzing communication fluctuations across the brain, this approach has been instrumental in studying complex neuropsychiatric disorders such as Frontotemporal Dementia (FTD) (19, 20) and schizophrenia (SCZ), enhancing our diagnostic capabilities and potential therapeutic interventions.

Therefore, in this Research Topic, we present a collection of articles that showcase recent advances in neuroimaging and non-invasive brain stimulation techniques and their application to the study of the brain's structural and functional organization.

Understanding the brain's structure and function is vital in the diagnosis and treatment of neuropsychiatric disorders. Advances in neuroimaging and NIBS techniques have enabled researchers to explore the underlying mechanisms of disorders such as depression, SCZ, anxiety and post-traumatic stress disorders (18, 21, 22). Identifying the neural circuits and networks involved in these disorders can lead to targeted interventions that aim to modulate brain activity and restore normal function (23). This deeper understanding has the potential to revolutionize the diagnosis and treatment of neuropsychiatric disorders, significantly improving the lives of millions of individuals worldwide.

These articles demonstrate the applications of neuroimaging in studying drug abuse, bipolar disorder (BP), dysmenorrhea, white matter lesions (WML), functional dyspepsia (FD), and SCZ. The meta-analysis of cocaine addiction shows how drug abuse affects the brain. The study on BP reveals the relationship between cerebral WML and the incidence of BP. The investigation of primary dysmenorrhea offers insights into the relationship between pain and the brain, while the exploration of differential brain responses to meal ingestion in FD patients provides a better understanding of this meal-induced syndrome. Finally, structural magnetic resonance imaging studies provide insights into the pathophysiology of SCZ.

Cocaine addiction causes significant changes in brain structure and function (24), affecting gray matter volume, white matter integrity, and neural activity, according to a meta-analysis by Dang et al. These findings suggest that drug addiction is a complex neurobiological disorder and not solely a behavioral problem. Identifying the specific brain regions and circuits impacted by cocaine addiction can help develop new treatments targeting these neural mechanisms (25). The study's implications are vital for the diagnosis and treatment of drug addiction.

Nyatega et al.'s study on BP found that individuals with the condition have altered striatal functional connectivity and structural dysconnectivity in the brain. These findings could serve as biomarkers for early detection and personalized treatment approaches for BP, advancing our understanding of neuropsychiatric disorders. The research provides insights into the structural and functional organization of the brain and has significant implications for improving the diagnosis and treatment of neuropsychiatric disorders (26, 27). In summary, this study's contributions could lead to more effective treatments and improve our understanding of BP.

Liu et al. studied the relationship between primary dysmenorrhea and brain activity changes, finding that patients with the condition have altered activity in the mesocorticolimbic pathway. This pathway is involved in pain processing and emotional regulation (28), suggesting that chronic pain conditions may be linked to changes in brain activity (29). The study's relevance to neuropsychiatric disorders is significant, as it highlights the importance of considering structural and functional changes in the brain when developing treatment plans for patients with chronic pain conditions (30). These findings provide valuable insights into the underlying mechanisms of chronic pain and may lead to more effective treatments (31).

Du et al. present a secondary analysis of data from a cross-sectional study investigating the non-linear correlation between the volume of cerebral WML and the incidence of BD. The study found that there is a positive and non-linear correlation between WML volume and BD risk, with the correlation being stronger when WML volume was < 6,200 mm^3^ (Du et al.). These findings provide valuable insights into the structural and functional organization of the brain in individuals with BD (32). The study's results may have important implications for the diagnosis and treatment of neuropsychiatric disorders, as they suggest that WML volume could be used as a biomarker for BD risk assessment.

Chen et al. used resting-state fMRI to investigate brain responses to meal ingestion in FD patients. They found abnormal connectivity in areas related to pain processing and emotional response networks, including the left postcentral gyrus, right precuneus, right middle frontal gyrus, anterior cingulate cortex, and right inferior frontal gyrus (Chen et al.). These findings provide insights into the structural and functional organization of the brain in FD patients and may have implications for the diagnosis and treatment of neuropsychiatric disorders involving visceral hypersensitivity and emotional dysregulation (33, 34). Overall, this study's contributions could lead to more effective treatments for patients with FD and related disorders.

Adamu et al.'s structural MRI study sheds light on the pathophysiology of SCZ, showing that individuals with the disorder have structural brain abnormalities linked to specific symptom clusters and cognitive impairments. The study also highlights the use of machine learning to identify patterns of brain structure associated with symptoms and impairments. The findings contribute to our understanding of the structural and functional organization of the brain in neuropsychiatric disorders and could improve diagnosis and treatment (35, 36). By identifying specific brain structure patterns associated with symptoms, clinicians may develop more targeted interventions for individuals with SCZ.

The articles in this Research Topic highlight the importance of continued research on the structural and functional organization of the brain and its potential impact on the diagnosis and treatment of neuropsychiatric disorders (37–41). The studies presented provide valuable insights into the complex relationship between drug abuse, BP, dysmenorrhea, WML, FD, and SCZ. These findings contribute to our understanding of the structural and functional organization of the brain in neuropsychiatric disorders and offer potential biomarkers for early detection and personalized treatment approaches (42–45). Additionally, the use of machine learning to identify brain structure patterns associated with symptoms and impairments could lead to more targeted interventions for individuals with psychiatric disorders (46–48). While the introduction of machine learning techniques, including deep learning, to the clinical field has significantly enhanced our understanding of diseases, the use of these techniques in diagnostics is often overlooked due to the “black box” phenomenon (49). This issue is especially conspicuous in the medical and psychiatric fields, where decisions regarding diagnoses and treatments bear direct and significant consequences for patients' lives. The “black box” problem refers to the obscurity of the inner workings of machine learning models. Despite their impressive predictive or analytical abilities, the lack of transparency in how these models arrive at their outputs often poses a significant challenge. This has led to the emergence of a specialized subfield known as explainable machine learning (50). This branch prioritizes creating models that, alongside delivering predictions or classifications, also provide clear explanations of how they reach these conclusions. By doing so, explainable machine learning attempts to solve the “black box” problem, promoting transparency and fostering greater trust in machine learning applications within the medical and psychiatric domains (49). Overall, these studies highlight the importance of considering both structural and functional changes in the brain when developing treatment plans for patients with neuropsychiatric disorders. In conclusion, continued research in this field could ultimately lead to more effective treatments and improved outcomes for individuals with these challenging conditions.

## Author contributions

MT, MD, and SB contributed to conception and design and wrote sections of the manuscript. MT wrote the first draft of the manuscript. All authors contributed to manuscript revision, read, and approved the submitted version.
